# Improving Relocations of Nursing Home Residents: A Participatory Design Approach

**DOI:** 10.1093/geroni/igaf122.012

**Published:** 2025-12-31

**Authors:** Annerieke Stoop, Charlotte Poot, Monique Caljouw

**Affiliations:** Tilburg University, Tilburg, Noord-Brabant, Netherlands; Leiden University Medical Center, Leiden, Zuid-Holland, Netherlands; Leiden University Medical Center, Leiden, Zuid-Holland, Netherlands

## Abstract

Relocations of nursing home residents within and between nursing homes occur for various reasons, including changing healthcare needs or renovation of outdated real estate. These relocations can have negative effects on physical and psychological functioning and mortality of residents and burn-out in staff. To prevent this, nursing homes deploy activities to support residents and staff during the relocation process. Yet, these activities are developed without involvement of relevant stakeholders and fail to address their needs and wishes. The objective of this study was to develop a prototype that aims to improve relocation experiences of residents, their families and staff. A participatory, user-centered design approach was used, in which residents, families and staff actively participated throughout the research. Context mapping was performed to explore current practices, relocation phases, impact on residents and staff, and identify best practices. Data were gathered from previous (Appreciative Inquiry) focus groups (n = 13) with stakeholders from sixteen nursing homes, complemented by knowledge sessions (n = 2) with researchers and social designers. Insights were used to generate design concepts which were translated into prototypes addressing key areas such as equal participation, space for emotions, feeling at home, effective communication and knowledge sharing. Prototypes were evaluated and tested with residents, families and staff in three iteration rounds and led to the development of a (comprehensive) relocation toolkit. This study demonstrates how participatory design supports the development of a tailored relocation intervention in which experiences and needs of residents, families and staff are reflected and contributes to reinforcement of research–practice partnerships.

